# 4-[(3-Chloro-2-methyl­phen­yl)imino­meth­yl]phenol

**DOI:** 10.1107/S1600536812043140

**Published:** 2012-10-20

**Authors:** B. C. Manjunath, M. M. M Abdoh, L. Mallesha, K. N. Mohana, N. K. Lokanath

**Affiliations:** a Department of Studies in Physics, Manasagangotri, University of Mysore, Mysore 570 006, India; b Department of Physics, Faculty of Science, An Najah National University, Nabtus West Bank, Palestinian Territories; c PG Department of Studies in Chemistry, JSS College of Arts Commerce and Science, Ooty Road, Mysore 570 025, India; d Department of Studies in Chemistry, University of Mysore, Manasagangotri, Mysore 570 006, India

## Abstract

In the title compound, C_14_H_12_ClNO, the dihedral angle between the aromatic rings is 39.84 (7)°. In th crystal, mol­ecules are connected by O—H⋯N hydrogen bonds into chains parallel to [001]. In addition, a C—H⋯π contact occurs.

## Related literature

For the bioactivity of the title compound, see: Corke *et al.* (1979 ▶); Gorrad & Manson (1989 ▶). For related structures, see: Jothi *et al.* (2012 ▶); Yaeghoobi *et al.* (2009 ▶). 
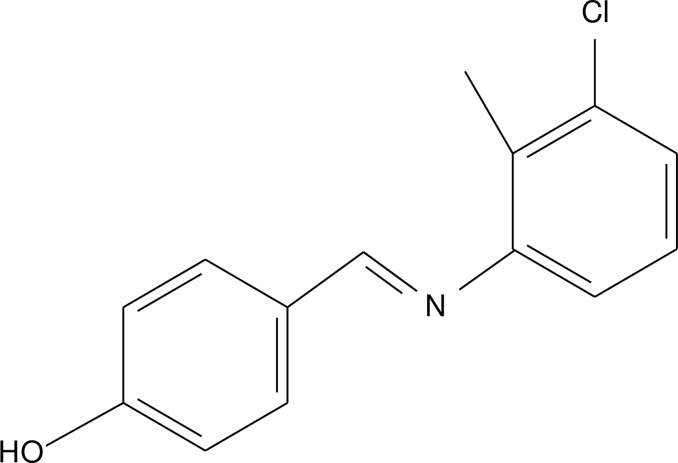


## Experimental

### 

#### Crystal data

C_14_H_12_ClNO
*M* *_r_* = 245.70
Orthorhombic, 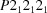

*a* = 7.5271 (9) Å
*b* = 12.4095 (15) Å
*c* = 12.5800 (14) Å
*V* = 1175.1 (2) Å^3^
*Z* = 4
Mo *K*α radiation
μ = 0.31 mm^−1^
*T* = 103 K
0.26 × 0.20 × 0.18 mm

#### Data collection

Oxford Diffraction Xcalibur Eos diffractometer
6050 measured reflections
2042 independent reflections
1856 reflections with *I* > 2σ(*I*)
*R*_int_ = 0.037

#### Refinement

*R*[*F* ^2^ > 2σ(*F* ^2^)] = 0.047
*wR*(*F* ^2^) = 0.123
*S* = 1.07
2042 reflections
155 parameters
H-atom parameters constrained
Δρ_max_ = 0.71 e Å^−3^
Δρ_min_ = −0.46 e Å^−3^

### 

Data collection: *CrysAlis PRO* (Oxford Diffraction, 2009 ▶); cell refinement: *CrysAlis PRO*; data reduction: *CrysAlis PRO*; program(s) used to solve structure: *SHELXS97* (Sheldrick, 2008 ▶); program(s) used to refine structure: *SHELXL97* (Sheldrick, 2008 ▶); molecular graphics: *Mercury* (Macrae *et al.*, 2006 ▶); software used to prepare material for publication: *publCIF* (Westrip, 2010 ▶).

## Supplementary Material

Crystal structure: contains datablock(s) global, I. DOI: 10.1107/S1600536812043140/kj2211sup1.cif

Structure factors: contains datablock(s) I. DOI: 10.1107/S1600536812043140/kj2211Isup2.hkl

Supplementary material file. DOI: 10.1107/S1600536812043140/kj2211Isup3.cml

Additional supplementary materials:  crystallographic information; 3D view; checkCIF report

## Figures and Tables

**Table 1:** Hydrogen-bond geometry (Å, °) *Cg* is the centroid of the C11–C16 ring.

*D*—H⋯*A*	*D*—H	H⋯*A*	*D*⋯*A*	*D*—H⋯*A*
O2—H2⋯N3^i^	0.84	2.05	2.854 (3)	160
C17—H17*C*⋯*Cg* ^ii^	0.98	2.73	3.649 (2)	157

Symmetry codes: (i) 
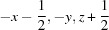
; (ii) 
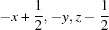
.

**Fig. 1.**
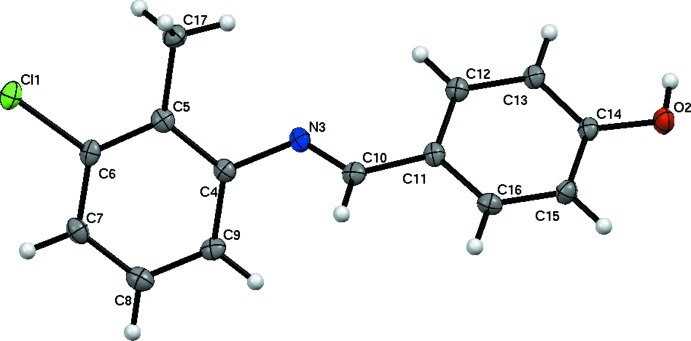
ORTEP diagram of the title compound with 50% probability ellipsoids.

**Fig. 2.**
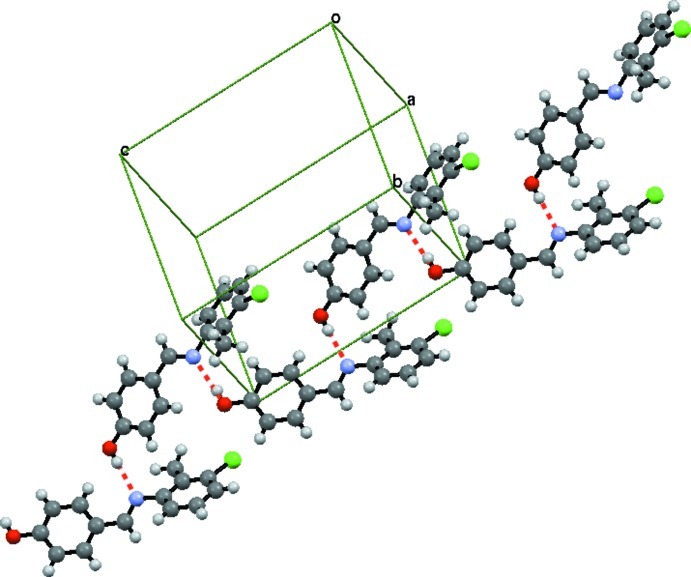
Packing diagram of the title compound, viewed along [1 - 1 0] direction. O—H···N hydrogen bonds are indicated by dashed lines.

## supplementary crystallographic information

### Comment

The investigation of microbial degradation by hazardous compounds such as anilines containing a methyl and a chlorosubstituent is of interest because of their lipophilic character and affinity to interact with DNA (Gorrad & Manson, 1989).

The *ORTEP* drawing of the title molecule is shown in Fig. 1. The 4-hydroxybenzylidene system is nearly planar and its geometry is similar to 4-chloro-2-[(*E*)-2-(4-methoxyphenyl)-ethyliminomethyl]phenol (Yaeghoobi *et al.*, 2009). The dihedral angle between the methylphenol and chloromethylphenylimino ring systems is 39.84 (7)°.

The molecules are connected by O—H···N interactions into chains along the [0 0 1] direction (Fig. 2). There is a weak contact of the type C—H···*π [1/2-x,-y,-1/2+z]* with a C···Cg distance of 3.649 (2) Å between the methyl group of the chloromethyl ring and the phenol ring.

### Experimental

Equimolar concentrations of 4-hydoxybenzaldehyde (0.003 mol) and 3-chloro-2-methylbenzenamine (0.003 mol) were refluxed for 5 h using methanol (25 ml) as solvent. The progress of the reaction was followed by TLC until the reaction was complete. The reaction product was cooled to 273 K. The precipitate was filtered and washed with diethyl ether. The residue was recrystallized from methanol. Brown single crystals were obtained.

### Refinement

In the absence of significant anomalous dispersion effects Friedel pairs have been merged. All the hydrogen atoms of the compound are fixed geometrically (O—H = 0.88 Å and C—H= 0.93–0.97 Å) and allowed to ride on their parent atoms.

### Crystal data

**Table:**

C_14_H_12_ClNO	*F*(000) = 512
*M**_r_* = 245.70	*D*_x_ = 1.389 Mg m^−^^3^
Orthorhombic, *P*2_1_2_1_2_1_	Mo *K*α radiation, λ = 0.71073 Å
Hall symbol: P 2ac 2ab	Cell parameters from 2042 reflections
*a* = 7.5271 (9) Å	θ = 2.3–30.6°
*b* = 12.4095 (15) Å	µ = 0.31 mm^−^^1^
*c* = 12.5800 (14) Å	*T* = 103 K
*V* = 1175.1 (2) Å^3^	Block, brown
*Z* = 4	0.26 × 0.20 × 0.18 mm

### Data collection

**Table:**

Oxford Diffraction Xcalibur Eos diffractometer	1856 reflections with *I* > 2σ(*I*)
Radiation source: fine-focus sealed tube	*R*_int_ = 0.037
Graphite monochromator	θ_max_ = 30.6°, θ_min_ = 2.3°
Detector resolution: 16.0839 pixels mm^-1^	*h* = −10→7
ω scans	*k* = −17→16
6050 measured reflections	*l* = −16→17
2042 independent reflections	

### Refinement

**Table:**

Refinement on *F*^2^	Primary atom site location: structure-invariant direct methods
Least-squares matrix: full	Secondary atom site location: difference Fourier map
*R*[*F*^2^ > 2σ(*F*^2^)] = 0.047	Hydrogen site location: inferred from neighbouring sites
*wR*(*F*^2^) = 0.123	H-atom parameters constrained
*S* = 1.07	*w* = 1/[σ^2^(*F*_o_^2^) + (0.0838*P*)^2^ + 0.0338*P*] where *P* = (*F*_o_^2^ + 2*F*_c_^2^)/3
2042 reflections	(Δ/σ)_max_ < 0.001
155 parameters	Δρ_max_ = 0.71 e Å^−^^3^
0 restraints	Δρ_min_ = −0.46 e Å^−^^3^

### Special details

**Table:**

Geometry. Bond distances, angles *etc*. have been calculated using the rounded fractional coordinates. All su's are estimated from the variances of the (full) variance-covariance matrix. The cell e.s.d.'s are taken into account in the estimation of distances, angles and torsion angles
Refinement. Refinement on *F*^2^ for ALL reflections except those flagged by the user for potential systematic errors. Weighted *R*-factors *wR* and all goodnesses of fit *S* are based on *F*^2^, conventional *R*-factors *R* are based on *F*, with *F* set to zero for negative *F*^2^. The observed criterion of *F*^2^ > σ(*F*^2^) is used only for calculating -*R*-factor-obs *etc*. and is not relevant to the choice of reflections for refinement. *R*-factors based on *F*^2^ are statistically about twice as large as those based on *F*, and *R*-factors based on ALL data will be even larger.

### Fractional atomic coordinates and isotropic or equivalent isotropic displacement parameters (Å^2^)

**Table:**

	*x*	*y*	*z*	*U*_iso_*/*U*_eq_	
Cl1	0.20725 (8)	0.07415 (5)	0.21487 (5)	0.0206 (2)	
O2	−0.2400 (2)	0.06296 (14)	1.08991 (14)	0.0200 (5)	
N3	−0.0186 (3)	0.11403 (16)	0.60025 (16)	0.0151 (5)	
C4	0.0219 (3)	0.16256 (18)	0.50001 (19)	0.0142 (6)	
C5	0.0934 (3)	0.09792 (18)	0.41844 (19)	0.0144 (6)	
C6	0.1173 (3)	0.1473 (2)	0.31955 (19)	0.0147 (6)	
C7	0.0749 (3)	0.2551 (2)	0.3006 (2)	0.0182 (7)	
C8	0.0060 (3)	0.3171 (2)	0.3831 (2)	0.0192 (7)	
C9	−0.0203 (3)	0.27089 (18)	0.4822 (2)	0.0165 (6)	
C10	0.0205 (3)	0.16597 (19)	0.68499 (19)	0.0157 (6)	
C11	−0.0336 (3)	0.13243 (19)	0.79125 (19)	0.0146 (6)	
C12	−0.1136 (3)	0.03193 (19)	0.81002 (19)	0.0162 (6)	
C13	−0.1794 (3)	0.00693 (19)	0.91011 (19)	0.0154 (6)	
C14	−0.1689 (3)	0.08211 (19)	0.99228 (18)	0.0148 (6)	
C15	−0.0820 (3)	0.18052 (19)	0.9758 (2)	0.0170 (6)	
C16	−0.0153 (3)	0.20481 (19)	0.8757 (2)	0.0161 (6)	
C17	0.1424 (3)	−0.01811 (19)	0.4369 (2)	0.0191 (7)	
H2	−0.28810	0.00190	1.09040	0.0300*	
H7	0.09280	0.28580	0.23230	0.0220*	
H8	−0.02280	0.39070	0.37150	0.0230*	
H9	−0.06730	0.31310	0.53850	0.0200*	
H10	0.08860	0.23010	0.67830	0.0190*	
H12	−0.12270	−0.01910	0.75400	0.0190*	
H13	−0.23160	−0.06160	0.92270	0.0180*	
H15	−0.06890	0.23030	1.03260	0.0200*	
H16	0.04360	0.27160	0.86450	0.0190*	
H17A	0.09600	−0.06240	0.37870	0.0290*	
H17B	0.09100	−0.04250	0.50440	0.0290*	
H17C	0.27200	−0.02500	0.43980	0.0290*	

### Atomic displacement parameters (Å^2^)

**Table:**

	*U*^11^	*U*^22^	*U*^33^	*U*^12^	*U*^13^	*U*^23^
Cl1	0.0193 (3)	0.0256 (3)	0.0169 (3)	−0.0005 (2)	0.0037 (2)	−0.0025 (2)
O2	0.0225 (9)	0.0216 (8)	0.0160 (8)	−0.0038 (7)	0.0057 (7)	−0.0002 (7)
N3	0.0108 (9)	0.0184 (9)	0.0160 (9)	−0.0002 (7)	0.0016 (8)	0.0019 (7)
C4	0.0096 (10)	0.0162 (10)	0.0168 (11)	−0.0014 (8)	0.0009 (9)	0.0013 (8)
C5	0.0083 (9)	0.0180 (10)	0.0170 (11)	−0.0006 (8)	−0.0017 (9)	0.0008 (8)
C6	0.0082 (9)	0.0213 (10)	0.0146 (10)	−0.0013 (8)	0.0015 (8)	−0.0017 (8)
C7	0.0150 (11)	0.0215 (11)	0.0180 (12)	−0.0024 (9)	0.0001 (9)	0.0043 (9)
C8	0.0147 (11)	0.0183 (11)	0.0246 (12)	−0.0002 (9)	−0.0011 (10)	0.0039 (9)
C9	0.0108 (10)	0.0173 (11)	0.0214 (12)	0.0014 (8)	0.0012 (9)	−0.0009 (9)
C10	0.0108 (10)	0.0171 (10)	0.0191 (11)	0.0005 (8)	0.0020 (9)	0.0000 (8)
C11	0.0104 (9)	0.0191 (10)	0.0144 (10)	0.0004 (8)	0.0002 (9)	0.0009 (9)
C12	0.0133 (10)	0.0189 (10)	0.0164 (11)	0.0002 (8)	−0.0002 (9)	−0.0003 (8)
C13	0.0121 (10)	0.0175 (10)	0.0167 (10)	−0.0003 (8)	0.0017 (9)	0.0002 (9)
C14	0.0121 (9)	0.0175 (10)	0.0148 (10)	0.0013 (8)	0.0016 (8)	0.0001 (8)
C15	0.0172 (11)	0.0178 (10)	0.0160 (11)	−0.0013 (9)	−0.0001 (10)	−0.0026 (8)
C16	0.0135 (11)	0.0151 (10)	0.0196 (11)	−0.0014 (8)	−0.0012 (9)	0.0008 (9)
C17	0.0185 (12)	0.0180 (11)	0.0207 (12)	0.0029 (9)	0.0033 (10)	0.0003 (9)

### Geometric parameters (Å, º)

**Table:**

Cl1—C6	1.737 (2)	C12—C13	1.388 (3)
O2—C14	1.361 (3)	C13—C14	1.395 (3)
O2—H2	0.8400	C14—C15	1.401 (3)
N3—C4	1.430 (3)	C15—C16	1.389 (4)
N3—C10	1.280 (3)	C7—H7	0.9500
C4—C9	1.399 (3)	C8—H8	0.9500
C4—C5	1.409 (3)	C9—H9	0.9500
C5—C6	1.398 (3)	C10—H10	0.9500
C5—C17	1.504 (3)	C12—H12	0.9500
C6—C7	1.396 (3)	C13—H13	0.9500
C7—C8	1.392 (3)	C15—H15	0.9500
C8—C9	1.386 (4)	C16—H16	0.9500
C10—C11	1.458 (3)	C17—H17A	0.9800
C11—C16	1.398 (3)	C17—H17B	0.9800
C11—C12	1.405 (3)	C17—H17C	0.9800
			
C14—O2—H2	109.00	C11—C16—C15	120.9 (2)
C4—N3—C10	118.2 (2)	C6—C7—H7	120.00
N3—C4—C5	118.9 (2)	C8—C7—H7	120.00
C5—C4—C9	121.1 (2)	C7—C8—H8	120.00
N3—C4—C9	119.8 (2)	C9—C8—H8	120.00
C4—C5—C6	116.6 (2)	C4—C9—H9	120.00
C4—C5—C17	121.7 (2)	C8—C9—H9	120.00
C6—C5—C17	121.7 (2)	N3—C10—H10	118.00
Cl1—C6—C5	119.71 (18)	C11—C10—H10	118.00
Cl1—C6—C7	117.42 (18)	C11—C12—H12	120.00
C5—C6—C7	122.9 (2)	C13—C12—H12	120.00
C6—C7—C8	119.2 (2)	C12—C13—H13	120.00
C7—C8—C9	119.7 (2)	C14—C13—H13	120.00
C4—C9—C8	120.6 (2)	C14—C15—H15	120.00
N3—C10—C11	123.8 (2)	C16—C15—H15	120.00
C10—C11—C16	119.1 (2)	C11—C16—H16	120.00
C12—C11—C16	119.0 (2)	C15—C16—H16	119.00
C10—C11—C12	121.8 (2)	C5—C17—H17A	110.00
C11—C12—C13	120.3 (2)	C5—C17—H17B	109.00
C12—C13—C14	120.2 (2)	C5—C17—H17C	109.00
O2—C14—C13	122.0 (2)	H17A—C17—H17B	109.00
O2—C14—C15	118.0 (2)	H17A—C17—H17C	109.00
C13—C14—C15	120.0 (2)	H17B—C17—H17C	109.00
C14—C15—C16	119.5 (2)		
			
C10—N3—C4—C5	138.4 (2)	C6—C7—C8—C9	−0.4 (3)
C10—N3—C4—C9	−45.7 (3)	C7—C8—C9—C4	0.0 (3)
C4—N3—C10—C11	171.9 (2)	N3—C10—C11—C12	8.7 (4)
N3—C4—C5—C6	175.1 (2)	N3—C10—C11—C16	−167.4 (2)
N3—C4—C5—C17	−5.2 (3)	C10—C11—C12—C13	−173.6 (2)
C9—C4—C5—C6	−0.8 (3)	C16—C11—C12—C13	2.5 (3)
C9—C4—C5—C17	178.9 (2)	C10—C11—C16—C15	173.2 (2)
N3—C4—C9—C8	−175.3 (2)	C12—C11—C16—C15	−2.9 (3)
C5—C4—C9—C8	0.6 (3)	C11—C12—C13—C14	1.0 (3)
C4—C5—C6—Cl1	179.21 (17)	C12—C13—C14—O2	176.5 (2)
C4—C5—C6—C7	0.5 (3)	C12—C13—C14—C15	−4.0 (3)
C17—C5—C6—Cl1	−0.5 (3)	O2—C14—C15—C16	−177.0 (2)
C17—C5—C6—C7	−179.2 (2)	C13—C14—C15—C16	3.5 (3)
Cl1—C6—C7—C8	−178.66 (18)	C14—C15—C16—C11	0.0 (3)
C5—C6—C7—C8	0.1 (3)		

### Hydrogen-bond geometry (Å, º)

Cg is the centroid of the C11–C16 ring.

**Table:**

*D*—H···*A*	*D*—H	H···*A*	*D*···*A*	*D*—H···*A*
O2—H2···N3^i^	0.84	2.05	2.854 (3)	160
C12—H12···O2^ii^	0.95	2.37	3.204 (3)	146
C17—H17*B*···N3	0.98	2.43	2.895 (3)	108
C17—H17*C*···*Cg*^iii^	0.98	2.73	3.649 (2)	157

Symmetry codes: (i) −*x*−1/2, −*y*, *z*+1/2; (ii) −*x*−1/2, −*y*, *z*−1/2; (iii) −*x*+1/2, −*y*, *z*−1/2.
